# Environmental Tobacco Smoke: Barnes et al. Respond

**DOI:** 10.1289/ehp.10233R

**Published:** 2007-09

**Authors:** Richard L. Barnes, Stanton A. Glantz, S. Katharine Hammond

**Affiliations:** University of California, San Francisco, San Francisco, California, E-mail: glantz@medicine.ucsf.edu; University of California, Berkeley, Berkeley, California

We are gratified that Jenkins does not dispute the central findings of our study (Barnes et al. 2006), namely, that *a*) the 16 Cities Study grew out of the tobacco industry’s plan to block any Occupational Safety and Health Administration (OSHA) standard on secondhand tobacco smoke; *b*) RJ Reynolds Tobacco was the originator of the 16 Cities Study and exercised substantial control of the research at all times; *c*) Jenkins et al. (1996) combined exposure data from restricted and unrestricted smoking workplaces and compared exposure data in an inappropriate manner that produced results the industry could cite to support its claim that workplace secondhand smoke (SHS) exposures were low compared with household exposures during its efforts to defeat indoor smoking restrictions; and *d*) a proper analysis of the data Jenkins presented indicates that smoke-free policies would halve the total SHS exposure of those living with smokers and virtually eliminate exposure for most others, supporting the need for smoke-free workplaces [and the polar opposite conclusion of Jenkins et al. (1996)]. [Compare Figures 1 and 2 of our article (Barnes et al. 2006)].

The disagreement appears to be in how transparent or opaque these facts were to the reader of Jenkins’ original article on the 16 Cities Study (Jenkins et al. 1996) and to OSHA.

In his letter, Jenkins ignores our Table 2 (Barnes et al. 2006), which contrasts the actual roles that RJ Reynolds and other agencies played in the design, conduct, and management of the 16 Cities Study compared with how these roles were described by Jenkins in his publications and direct testimony. We did not say that he did not disclose that he was working for the tobacco industry; we presented evidence that the disclosures in his articles did not completely reflect the role that the industry played in conceiving of and controlling the study. In addition, lengthy cross-examination of Jenkins during the OSHA hearings was required to reveal the extensive involvement of RJ Reynolds, and that revelation was incomplete (OSHA 1995).

We also would like to address a few other small points. First, far from “deliberate selection of data,” we followed standard snowball methodology (Malone and Balbach 2000) for searching the industry documents; we identified > 500 relevant industry documents, as well as court records and the published literature, as a basis for our article (Barnes et al. 2006). We did analyze the full public 16 Cities data set when preparing our article, but we did not cite it because we were able to present our analysis based on summary results from the published articles (Jenkins and Counts 1999; Jenkins et al. 1996). We did not mention the Dunn–Wiley trial (Dunn and Wiley et al. v. RJR Nabisco Holdings Corp. et al. 1993) in Indiana because the ruling was on a motion to strike Jenkins’ testimony because of procedural issues relating to disclosure of expert witness testimony in advance of trial, not a challenge to the conduct of the 16 Cites Study, as made in the Brion case (Dunn and Wiley et al. v. RJR Nabisco Holdings Corp. et al. 1998).

Nothing in Jenkins’ letter contradicts our conclusion that he and his colleagues presented the data from the 16 Cities Study in a way that conformed to the stated objective of the Tobacco Institute’s “OSHA Projects” to “encourage adoption of a ventilation standard and to discourage adoption of a smoking ban or of a standard that requires separate ventilation for areas where smoking is allowed” (Tobacco Institute 1991). Indeed, as noted above—and unchallenged by Jenkins—a proper presentation of the 16 Cities data [Figures 1 and 2 of Barnes et al. (2006)] shows that employees in “smoking workplaces” have significant levels of SHS exposure and that smokefree workplaces substantially reduce overall exposure to SHS. This conclusion remains important because the tobacco industry and its allies still rely heavily on the 16 Cities Study in opposing regulation of SHS exposures.
